# Structural and Technological Aspects of Improving the Accuracy of Worm Gears in the Processes of Design, Manufacturing, and Assembly

**DOI:** 10.3390/ma19091712

**Published:** 2026-04-23

**Authors:** Wojciech Kacalak, Jacek Ponomarenkow, Katarzyna Tandecka, Maciej Majewski, Zbigniew Budniak

**Affiliations:** 1 Department of Engineering and Informatics Systems, Faculty of Mechanical Engineering and Energy, Koszalin University of Technology, 75-620 Koszalin, Poland; wojciech.kacalak@tu.koszalin.pl (W.K.); j.ponomarenkov@gmail.com (J.P.); zbigniew.budniak@tu.koszalin.pl (Z.B.); 2 Faculty of Computer Science, Gdynia Maritime University, Morska 81-87, 81-225 Gdynia, Poland; m.majewski@wi.umg.edu.pl

**Keywords:** worm gears, kinematic accuracy, backlash minimization, assembling optimization

## Abstract

**Highlights:**

- Optimization of design, grinding, and assembly has improved the precision of the worm gear system.
- The implementation of an axially flexible worm led to a reduction of backlash without taking apart the gearbox for modification.
- Assembly optimization lowered the range of backlash from 20 μm to 7–10 μm.
- The backlash standard deviation was decreased to about 1.8 μm after assembly optimization.

**Abstract:**

This paper discusses ways to improve the kinematic accuracy of worm gears in batch production. Worm gears are used in applications where high positioning accuracy, uniform motion and vibration damping is required. The paper focuses on three main methods: design changes, manufacturing process improvements and assembly optimization. Design changes aim to reduce dimensional and shape deviations of worm and worm wheel surfaces, with focus on the axially flexible worm design, which allows for minimizing backlash without disassembly. Manufacturing refinements, especially helical surface grinding, improve gear accuracy and durability. The developed algorithm for small batch production allows for selecting components based on specific criteria and thus improves overall production quality. With respect to optimization, the backlash ranges between 2 and 22 micrometers, meaning that its entire range is 20 micrometers. However, after optimizing, the backlash range falls between 7 and 10 micrometers, depending on the criterion for optimization, which amounts to about 50 to 65 percent of the initial range. The methods and algorithms are universal and can be used in small batch and large scale production. They bring economic benefits by reducing production costs and downtime through easy backlash adjustment.

## 1. Introduction

In many modern devices, very high positioning accuracy of measurement and machining systems is required. This is particularly relevant to mechanisms for precise movements and other systems demanding uniform motion and good vibration damping under variable load conditions. Worm gears are widely used in kinematic systems where high gear ratios, high motion transmission accuracy, and self-locking capabilities are needed. They are most commonly employed in positioning systems in technological devices and measurement systems [1,2]. Additionally, worm drives are found in measurement heads, rotary tables, steering gearboxes in cars, worm reducers, and control systems for the drives of ships, vehicles, and military equipment. Numerous research centers are working on improving the accuracy of these gear systems. Three main methods contribute to this goal. The first involves modifications to the construction [3], covering design aspects to enhance accuracy [4,5], including patented solutions by the authors [6]. The second method for improving accuracy is refining the manufacturing processes of gear elements [7,8], especially the precision shaping of the worm gear’s helical surfaces and the geometrically coupled gear teeth surfaces of the worm wheel [9,10]. Improving the manufacturing processes of worm gears [11] has resulted in a very high level of quality and increased durability due to the improved properties of the interacting surfaces [12,13]. These effects, along with the typical features of the discussed gear systems, such as high stiffness and load capacity, contribute to their steadily increasing range of applications. Dedicated manufacturing methods have also been developed [14]. The third method for enhancing gear accuracy pertains to assembly, involving the selection of gear elements in such a way that the overall series’ accuracy, evaluated based on the gear with the lowest accuracy, reaches the highest values. In many cases, the second (technological) and the third methods for improving gear accuracy enable achieving the desired precision level of the components. Increasing kinematic accuracy does not imply backlash-free operation, as the components still exhibit certain, though smaller, deviations in the dimensions and shapes of the interacting teeth surfaces. Moreover, these deviations increase due to wear [15] during gear operation.

In addition to improvements in geometry and technology, it should be kept in mind that the performance of worm gears is also influenced by lubrication conditions and the use of lubricants with modern additives, including modern additives. These approaches can help reduce the effects of unavoidable geometric deviations by improving load-carrying capacity, reducing friction, and decreasing vibration. However, they do not remove the need for high geometric and assembly accuracy, because deviations in the dimensions and positions of mating parts remain one of the main causes of backlash and other operational problems. In worm gears, high sliding speeds in the contact zone make the system particularly sensitive to geometric accuracy and misalignment. Such deviations can lead to deteriorated motion, increased vibration, and reduced efficiency. Therefore, improving kinematic and geometric accuracy should be seen not only as a geometric issue, but also as an important condition for better gear performance. Traditional approaches to sensitivity analysis usually assume simple principles, dealing with each deviation separately and assuming linear dependencies between the elements of deviations. Moreover, they do not take into account non-linear interdependencies between the mentioned deviations, which become especially important in cases when high loads are combined with sliding velocity, which is inherent to high-power worm gears. Additionally, such methods neglect variations in parameters characteristic of practical production operations, particularly small-batch manufacturing.

**Figure 1:**
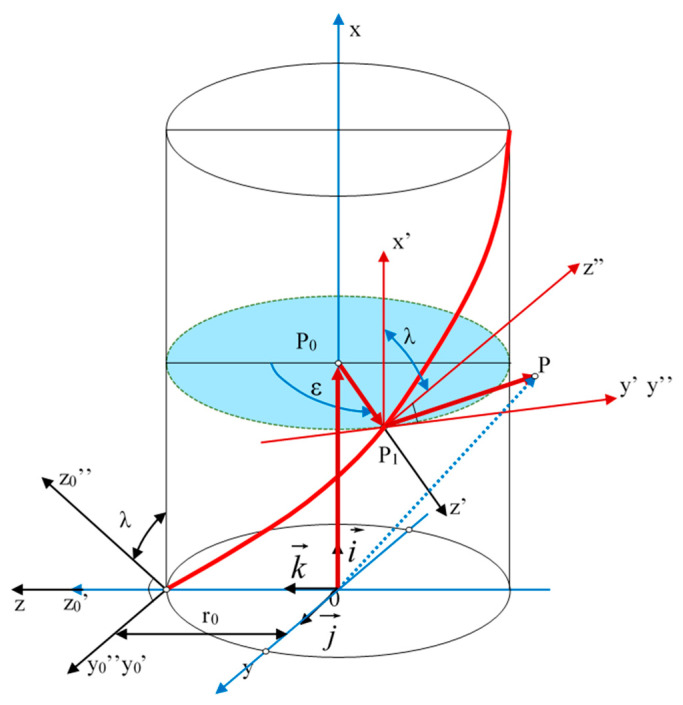
The general form of the helical surface; Equation (1).

An effective way to ensure higher accuracy is to modify the design [16] of the worm, worm wheel, or their assembly to allow for minimizing side backlash through appropriate regulation, either adaptive or periodically introduced without disassembling the gear [17]. The analysis of various design solutions in this area has been presented in works [18,19]. In particular cases, design modifications are also made due to movement resistance [20,21] or features of the meshing zone [22]. In designing new solutions, methods for creative problem-solving in construction, utilizing antipatterns, and simulating surface shaping processes using CAD systems [23,24] and mathematical analyses [25] can be useful. In addition, the assessment of the accuracy and quality of the surfaces can be achieved using the stereometric characteristics of technical surfaces [26] and modeling the process of formation of the helical surfaces [27]. In the case of efficient worm gears, thermal and backlash aspects also have an important role to play, especially in different operating regimes [28,29]. The most critical requirements for helical surface shaping processes concern the high accuracy of the lead, as this determines the positioning accuracy of the moved elements. The load-bearing capacity and durability of the gear depend on the accuracy of the helical surface profiles of the interacting gear elements, as well as the stereometric properties of the surfaces and the physical properties of the surface layer. The accuracy of the profile directly influences the highest frequency harmonic components of the gear backlash. Helical surfaces can be of various types. They may be surfaces generated by helical lines situated on a cylinder, cone, or any other surface. Among the surfaces formed by lines located on a cylinder, rectilinear and non-rectilinear surfaces can be distinguished. Non-rectilinear surfaces do not have a straight generating line in any cross-section by an arbitrary plane. Helical surfaces can vary in the shape of their profiles, which can be defined in different cross-sections. Some surfaces are not defined by specifying their shape (profile) but rather by specifying the tool profile and type used for shaping them, while others have a shape directly defined by the profile itself. The profiles in specific cross-sections of helical surfaces after shaping the machined elements depend not only on the nominal geometric parameters (structurally defined) of the helical surfaces but also on the shape, dimensions, and position of the tool. Precise helical surfaces of worms with hardness above 36 HRC are shaped by grinding. The number of different types of helical surfaces is not limited. However, it is important to note that to fully define it, the geometric parameters of the tool must also be specified. Additional modeling and simulation studies of worm gear meshing and contact have also been reported in the literature [30,31], while recent work has further examined adaptive backlash minimization in precision positioning systems [32]. Similar optimization approaches are also known from related gear design problems [33]. For practical reasons, standardization is per-formed to establish standardized types of surfaces. The distinguishing feature is the shape of the profile in a specific flat cross-section, which identifies only rectilinear surfaces. The distinguishing feature of non-rectilinear surfaces is the shape of the tool. However, it is important to note that to fully define it, the geometric parameters of the tool must also be specified. The general equation of the helical surface (1) has particular cases obtained by considering the position of the plane in which the helical surface profile is defined, i.e. (Figure 1), the values of the angle λ and the distance *r_0_* when λ = 0, and the form of the profile functions ψ(u) and φ(u) in this plane, which are implicit dependencies for non-rectilinear surfaces.

(1) $$\begin{array}{c} \vec{OP}=\left[\phi \left(u\right)\cos\lambda +P\frac{\varepsilon }{2\pi }\right]\vec{i}+\left[\phi \left(u\right)\sin\lambda \sin\varepsilon +\psi \left(u\right)\cos\varepsilon +r_{0}\sin\varepsilon \right]\vec{j}+\\ +\left[\phi \left(u\right)\sin\lambda \cos\varepsilon -\psi \left(u\right)\sin\varepsilon +r_{0}\cos\varepsilon \right]\vec{k} \end{array}$$

According to recent studies, improved lubrication approaches that employ nano-additives may help alleviate the effect of meshing errors on worm gears, thus improving the load-carrying ability of such gearboxes [34,35].

In spite of the well-recognized importance of accuracy in the design and manufacture of worm gears, there is still no integrated approach that takes into account the geometric, technological, and assembly related factors contributing to inaccuracy under practical manufacturing conditions. Current practice often considers design parameters, machining accuracy, and assembly selection separately, without fully addressing the problem of backlash and kinematic inaccuracy in the final gear. Therefore, the main goal of this paper is to develop a general approach that includes the analysis of geometric and technological causes of inaccuracies in worm and worm wheel components, the use of constructive solutions for adaptive backlash reduction, and the selection of components during assembly in small-series production in order to minimize the variation in backlash parameters across the whole production series. It sets itself apart from other methods since it studies the combined effects of geometric, technological, and assembly considerations, as opposed to looking at each one separately. This results in an evaluation that is much closer to reality, and it allows for better quality control in the manufacture of gears. This approach combines geometric, technological and assembly related aspects within one system, together with optimization of component selection in small-batch production. As a result, it allows a more realistic assessment of backlash variation and its effective reduction.

## 2. Materials and Methods

**Figure 2:**
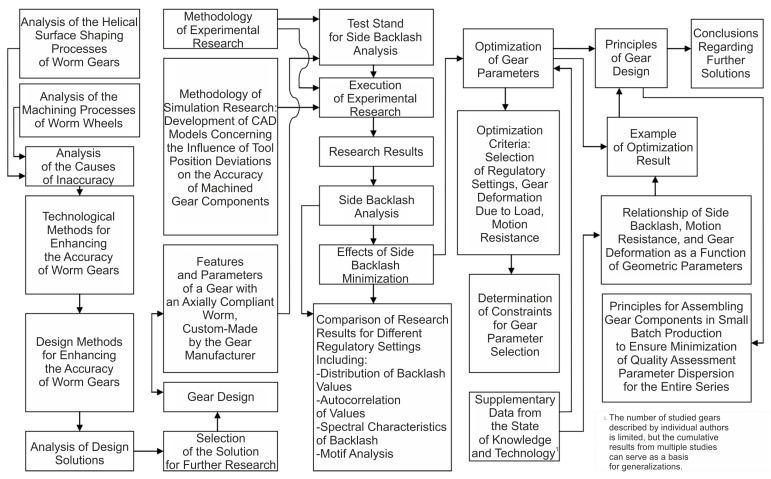
Comprehensive analysis of backlash minimization issues.

This publication presents a methodology for enhancing the kinematic accuracy of worm gears with an innovative design that ensures adaptive backlash minimization. The scope of research leading to a comprehensive analysis of backlash minimization problems included several stages. The framework for the proposed strategy is depicted in Figure 2. It highlights the relationship between the various stages of design, manufacture, and assembly. This flow represents the nature of an iterative process and how different variables affect the final backlash value.

**Figure 3:**
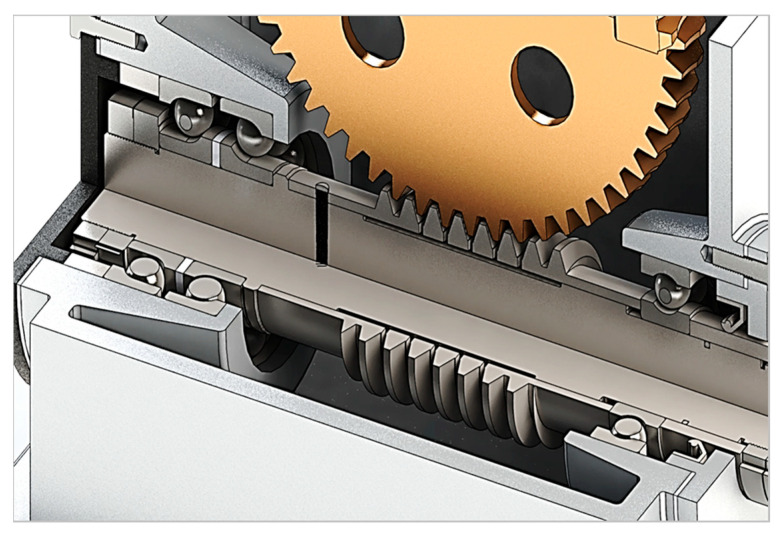
General visual representation of the worm gear arrangement considered in the study, showing the worm, the worm wheel, and the central compliant zone of the worm.

In the investigated worm gears, the worms are mounted on a shaft and have a cut in the middle part of the thread root (Figure 3). This design makes the worm axially compliant in this central zone, allowing for the reduction in backlash in the gear through slight axial compression without disassembly. The first part of the paper presents selected results from the study of the statistical characteristics of backlash. Figure 3 below shows the overall visualization of the worm gear system analyzed in this research. It illustrates the respective locations of the worm, the worm gear, and the compliant region of the worm resulting from the thread root cutting process. The figure is intended to support the understanding of the proposed design approach, but it is not intended to be an engineering diagram.

After analyzing technological and design methods for minimizing side backlash in worm gears, the subsequent part of this publication presents analyses regarding various criteria for the optimal assembly of precision worm gears in small batch production. It is considered that the gear components—the housing, worm, and worm wheel—manufactured within specified accuracy tolerances, exhibit differences in shape and dimension deviations. As a result of assembling gears composed of elements with varying accuracy without applying optimization procedures, the resulting set of gears can have significantly varied kinematic deviation or side backlash. The developed optimization procedures aim to minimize the differences in accuracy among individual gears within the entire production series.

### 2.1. Remarks on Technological Methods for Enhancing the Accuracy of Worm Gears

**Figure 4:**
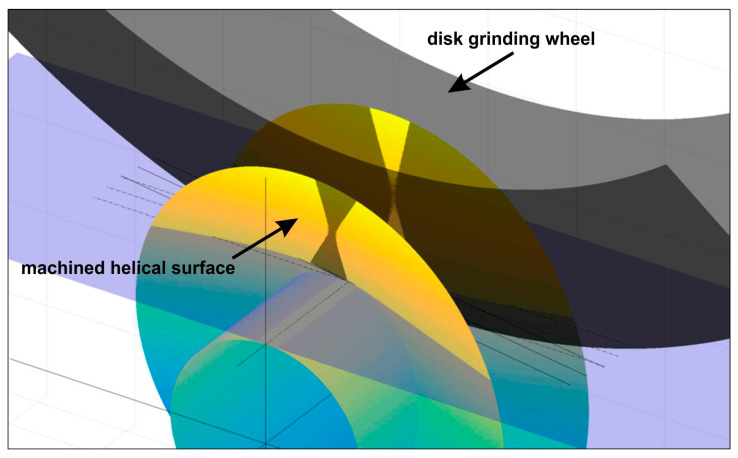
The shape and position of the machining zones during grinding of the cone-derived helical surface of the worm.

**Figure 5:**
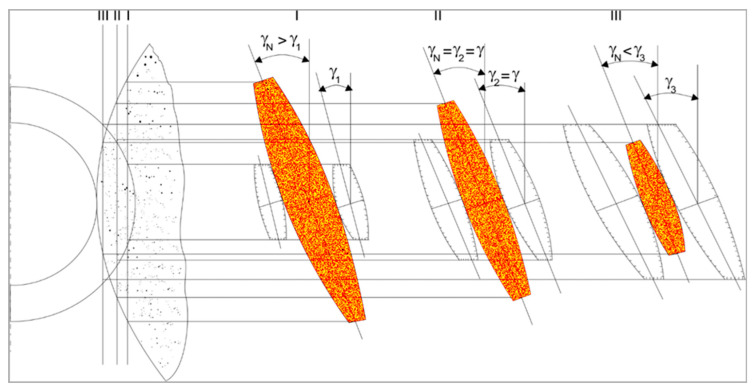
Diagram illustrating the shaping of the helical surface profile in the disk grinding process, including selected cross-sections (I, II, III) and successive positions of the grinding wheel [18].

The accuracy of worm gears significantly depends on the precision of machining the interacting helical surfaces of worm threads and worm wheel teeth. The most critical requirements concern the high accuracy of the worm lead and the pitch of the worm wheel teeth. This precision determines the positioning accuracy of the moved elements, while the profile accuracy affects the load-bearing capacity and durability of the gear. The technical observations presented here serve as the basis for understanding the causes of errors that are then analyzed when modeling the accuracy of the helical surface, as well as their influence on backlash. The helical surfaces of worm threads with a hardness below 36 HRC can be ground or machined using turning or milling operations, employing special tools and small cutting depths. Accurate helical surfaces with a hardness above 36 HRC require grinding. Grinding helical surfaces, considering the shape and position of the machining zone (Figure 4), is a process that necessitates the proper selection of grinding wheel characteristics, methods for shaping their surfaces, and ensuring high positioning accuracy of the movable elements in the technological system. Recommendations for assessing the state of the working surface of abrasive tools, their wear, and the methodology for evaluating the geometric structure and quality of ground surfaces can be utilized in this context. Helical surfaces are most commonly ground using disk grinding wheels. Finger, ring, or cup wheels can also be used, although using finger wheels severely limits grinding efficiency due to their low rigidity. Additionally, the small active surface area results in low shape durability. Cup and ring wheels do not allow simultaneous grinding of both sides of the groove, which eliminates their production utility. Disk grinding wheels are characterized by high rigidity, have a large active surface area, and enable simultaneous machining of both sides of the groove. This allows for high grinding efficiency but requires solving the problems of shaping the grinding wheel profile to match the specific helical surface shape. The helical surface shape is then the envelope of the tool’s shape in its relative helical movement concerning the machined surface (Figure 5). The helical surface shaped in the grinding process with a disk grinding wheel is the envelope of the tool’s active surface moving helically relative to it. Such a helical surface, in the general case, is not rectilinear. The mathematical relationships describing the geometric features of axial or normal profiles of helical surfaces are complex, especially for cone-derived and torus-derived surfaces. Occasionally, deviations from the proper nominal grinding wheel profile for grinding a specific helical surface may be acceptable if the profile error of the helical surface caused by this deviation is small compared to the permissible profile deviation, and the profile curvature increases slightly. To define the geometric features of the helical surface ground with a disk grinding wheel, it is necessary to specify: the nominal profile and dimensions of the tool, as well as its position relative to the axis of the machined surface. This method of definition arises from the fact that the helical surface shape and its profile in a specific cross-section depend on both the active surface profile of the tool and its diameter, as well as the position of the tool axis relative to the axis of the machined surface, for example, an inclination at an angle different from the lead angle of the helical line of the machined surface on the pitch cylinder. Figure 5 shows the concept of forming the helical surface by grinding process. The orange zones depict the positions of the wheel of the grinding device used at three different cross-sections (I, II, and III), showing the effect of the orientation of the axis of the grinding device on the profile formed on the surface of the helix. Hence, the figure enables an understanding of the geometry of the grinding wheel relative to the surface of the worm gear.

The profiles of helical surfaces most commonly used in engineering are standardized. The nominal profile of the helical surfaces of most threads is a straight line in the axial section. Standardized nominal profiles of worm helical surfaces are straight for:

- Archimedean worm A, whose nominal section is an axial section, and the nominal profile of the tooth is a straight line.
- Normal convolute worm N, whose nominal section is a normal section, and the nominal profile of the tooth is a straight line.

Curvilinear profiles are characteristic of:

- Axial-arc worm AR, whose nominal profile is a circular arc in the axial section.
- Normal-arc worm NR, whose nominal profile is a circular arc in the normal section.
- Involute worm E, which is a cylindrical involute gear.

Additionally, helical surfaces of worms can be defined by the nominal profile of a disk tool (these surfaces are non-rectilinear):

- Cone-derived worm K, whose nominal section is a normal section, and the nominal profile of the disk tool is a straight line.
- Toroidal-derived worm KR, whose nominal section is a normal section, and the nominal profile of the disk tool is a circular arc.

Other profiles can also be adopted, but this complicates the selection of tools for machining worm wheel teeth, as the profiles of the worm thread and the worm wheel teeth must have a specific geometric association. Justified profile modifications can be used when minor deviations from the nominal tool profile do not significantly alter the working conditions of the worm and worm wheel. In such cases, the worm and the worm gear milling cutter should be ground under identical conditions.

### 2.2. Mechanisms of Cumulative Effects of Inaccuracies

Considering the dimensional and form inaccuracies of ground worm helical surfaces and worm wheel teeth, it is not sufficient to determine only the measures of location and variability of the statistical set of results for a single realization of the process. What is important is not so much the distribution of results obtained for a completed batch of products, but rather the distribution of results for all possible process realizations.

In the present analysis, it was assumed that these distributions result from the combined effects of multiple causes of deviations.

The problem of selecting an appropriate distribution model for the analyzed characteristic in the general population cannot be solved solely by searching for a model that fits a given dataset. An analysis of the mechanism responsible for the occurrence of a specific dimensional or form deviation is indispensable. This reduces the probability of inconsistency between the adopted model and the results obtained in subsequent realizations, especially under changed operating conditions.

Changes in the condition of the active grinding wheel surface cause dimensional and form deviations to arise from processes that cannot be regarded as stationary. As a consequence of process non-stationarity, the overall distribution of these deviations differs from the instantaneous distribution and depends on both the length of the observation interval and the starting moment of data acquisition.

Based on analyses of numerous precision grinding processes, a methodological procedure was formulated, comprising the following sequence of research steps:

1. Evaluation of the causes of deviations and the locations of their occurrence within the technological system.
2. Analysis of the statistical characteristics of the causes of deviations and assessment of whether the deviation:
  - Results from a stationary or a non-stationary random process;
  - Contains a non-random component that is either constant or time-varying;
  - Is characterized by a high coefficient of variation.
3. Analysis of the effect of the considered cause on the occurrence of specific inaccuracy features, in particular an assessment of:
  - The transmission characteristics of random and deterministic signals through the technological system.
  - The probabilistic characteristics of the system response to excitation constituting the cause of the deviation.
4. Analysis of the mechanism of accumulation of the effects of multiple diverse causes on the resulting deviation distribution.

A deterministic analysis of the causes of deviations in the position of the tool surface relative to the machined surface is usually sufficient to identify the main causes of inaccuracy of the machined part. However, it does not make it possible to determine the influence of various causes on the nature and degree of dispersion of the values of the analyzed inaccuracy characteristic.

A dimensional or form deviation is often the result of the action of many factors of minor influence, none of which is dominant. In such a case, determining the cumulative effects of multiple influences makes it possible to infer the existence of specific causal relationships.

The mechanisms of the accumulation of inaccuracy effects may be described by an arithmetic sum, a geometric sum, a product, a set of extreme values, or a more complex function. For a process regarded as stationary, the following model cases can be distinguished:

- The resulting distribution is the distribution of the sum of random variables, with one or several factors having a decisive influence.
- The distribution is the sum of many factors, none of which dominates the others.
- The distribution of the analyzed inaccuracy characteristic is the distribution of the geometric sum of random variables.
- The dimensional or form deviation, as a random variable, depends on the product of a large number of other variables, or the main cause of the deviation results from the action of a multiplicative mechanism.
- The resulting distribution is the distribution of the scalar or vector product of random variables.
- The distribution of the analyzed inaccuracy characteristic is the distribution of extreme values of the component effects.

If none of the many causes of inaccuracy dominates the others, and these variables are not highly dependent, then the distribution of the sum of effects is close to the normal distribution.

In the grinding of worm helical surfaces and the hobbing of worm wheel teeth, it may be assumed that deviations are formed partly as a result of summation of elementary geometric effects. Even if the normal distribution is a consequence of the laws governing the dispersion of the values of the analyzed inaccuracy characteristic, its adequacy for describing a dataset decreases with increasing distance from the mean value. In the case of a small sample size, this may lead to serious errors.

A frequently encountered case of a multiplicative mechanism of deviation formation during grinding is the division of stock allowance into multiple passes. Each pass reduces the dimensional or form deviation randomly, in proportion to its value. The value of the deviation after the *n*-th pass is equal to the product of the deviation before that pass (*n* − 1) and a random factor representing the reduction in the deviation.

The resulting random variable, and in this particular case the resulting dimensional or form deviation, when the number of spark-out passes is large, is the product of a considerable number of other variables, and examining and describing each of them individually is impossible.

However, it is possible to determine the resulting distribution provided that the conditions associated with the central limit theorem are satisfied. In that case, the dispersion of deviation values is described by the log-normal distribution.

In the assessment of inaccuracies of ground elements, it often happens that quality is determined by the largest dimensional or form deviation. In worm gear elements, the principal inaccuracy characteristic of the worm may be the maximum value of the cumulative pitch deviation, whereas in worm wheels it may be the maximum deviation of the sum of any number of pitches.

In the above-mentioned cases, the maximum value xmx_mxm among nnn random variables x_1_, …, x_n_ is sought. If the variables x_i_ satisfy the assumptions of independence and identical distribution, then the distribution of the values x_i_ has a relatively small effect on the distribution of the variable x_m_ = max(x_i_). For form deviations of abrasively machined surfaces, the above assumptions are satisfied more often than for surfaces machined by other methods.

The Type I extreme value distribution is obtained under the assumption that the probability of occurrence of unboundedly increasing values of the variables x_i_ decreases exponentially. This means that the probability density function takes the form:

(2) $$f\left(x\right)=\frac{d(g\left(x\right))}{dx}e^{-g(x)}$$

where g(x) is an increasing function of the variable.

The probability density function of the maximum values then takes the form:

(3) $$f\left(x_{m}\right)=\alpha e^{-\alpha \left(x_{m}-u\right)-e^{-\alpha \left(z_{m}-u\right)}}$$

where u is the mode of the distribution of the variable x_m_, whereas α is a measure of dispersion. The constants 0.577 and 1.645 correspond to the Euler–Mascheroni constant and the variance of the Type I extreme value (Gumbel) distribution, respectively. The expected value and variance are then expressed by the following approximate relationships:

E(x_m_) = u + 0.577 α^−1^ (4)

D^2^(x_m_) = 1.645 α^−2^ (5)

and the skewness coefficient is equal to 1.1396.

The Type II limiting distribution of the largest values occurs when the random variable is bounded below by zero and unbounded above. In that case, the probability density functions of the value x and of the maximum values x_m_ take the following forms:

(6) $$f\left(x\right)=\beta kx^{-(k+1)},\;x\geq 0$$

(7) $$f\left(x_{m}\right)=ku^{k}{x_{m}}^{-(k+1)}e^{-{\left(\frac{u}{x_{m}}\right)}^{k}},\;x_{m}\geq 0$$

The relationship between the Type II and Type I distributions is the same as that between the log-normal and the normal distributions. If x_m_ has a Type II distribution, then ln(x_m_) has a Type I distribution. In another case, the Type III limiting distribution of the largest values corresponds to random variables bounded above and is known as the Weibull distribution.

### 2.3. Mathematical Modeling of the Geometric Properties of the OUPN Process System

**Figure 6:**
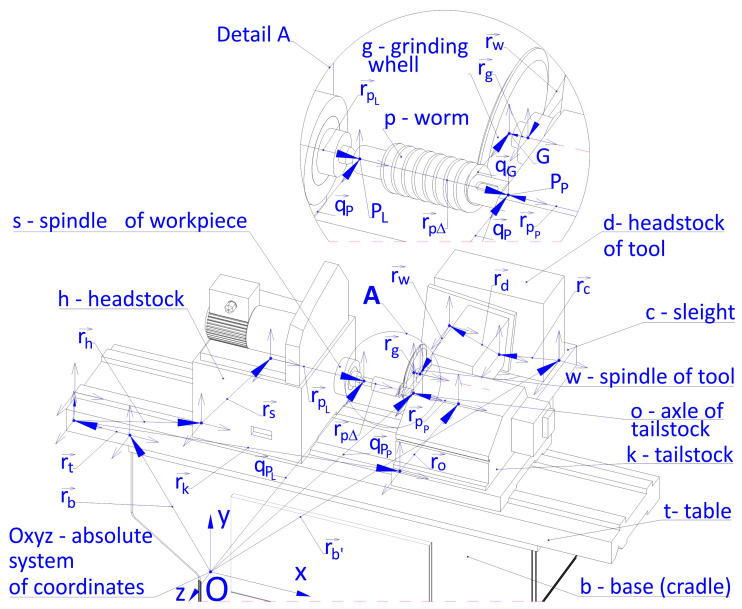
OUPN process system.

The mathematical model of the machining system comprises relations between the parameters defined in local systems of coordinates presented in Figure 6, which reflects the mutual geometric and kinematic relationships between the basic sets of the machine tool, starting from the base (bed) b, grinder table t, headstock of workpiece h, workpiece spindle s, tool headstock sled c, abrasive wheel headstock d, abrasive wheel spindle w, abrasive wheel g, knight k, knight axis o and workpiece p.

The positioning of the workpiece fastened in lathe centers (base points P_L_ and P_P_) in an absolute coordinate system Oxyz, is determined with vectors ${\vec{q}}_{P_{L}}$ and ${\vec{q}}_{P_{p}}$:

(8) $${\vec{q}}_{P_{L}}={\vec{r}}_{b}+{\vec{r}}_{t}+{\vec{r}}_{h}+{\vec{r}}_{s}+{\vec{r}}_{P_{L}}\cdot {\vec{q}}_{P_{p}}={\vec{r}}_{b}+{\vec{r}}_{t}+{\vec{r}}_{k}+{\vec{r}}_{o}+{\vec{r}}_{P_{p}}$$

The position of the grinding wheel (point G—start of the local coordinate system of the tool) in an absolute coordinate system Oxyz is determined with vector ${\vec{q}}_{G}$:

(9) $${\vec{q}}_{G}={\vec{r}}_{b}+{\vec{r}}_{c}+{\vec{r}}_{d}+{\vec{r}}_{w}$$

In a general case, the value of the closing link ${\vec{r}}_{P_{∆}}$ of the spatial dimensional chain of the OUPN system, which determines the positions of the worm machined, in an absolute system of coordinates Oxyz is calculated based on the following formula:

(10) $${\vec{r}}_{P_{∆}}={{\vec{q}}_{P}}_{L}-{{\vec{q}}_{P}}_{p}$$

where ${{\vec{q}}_{P}}_{L}$ i $-{{\vec{q}}_{P}}_{p}$ are the vectors that determine the position of points P_L_ and P_P_, which determine the position of the workpiece p fastened in w lathe centers, in relation to the global system of coordinates Oxyz.

The deviation of the angular position of the axis of the workpiece $\delta_{P_{∆}}$, which is the result of the deviation of the axis of the knight in relation to the spindle axis, as a link that closes the spatial angular dimensional chain, may be calculated from the following formula:

(11) $$\delta_{P_{∆}}=arccos\frac{{\vec{r}}_{s}\cdot {\vec{r}}_{o}}{\left|{\vec{r}}_{s}\right|\cdot \left|{\vec{r}}_{o}\right|}$$

where ${\vec{r}}_{s}\cdot {\vec{r}}_{o}$ is the scalar product of the vectors ${\vec{r}}_{s}$ i $\cdot \;{\vec{r}}_{o}$ and $\left|{\vec{r}}_{s}\right|$ i $\cdot \;\left|{\vec{r}}_{o}\right|$ are the lengths of vectors ${\vec{r}}_{s}$ i $\cdot \;{\vec{r}}_{o}$.

#### 2.3.1. Mathematical Model of the Helical Surface of the Worm

**Figure 7:**
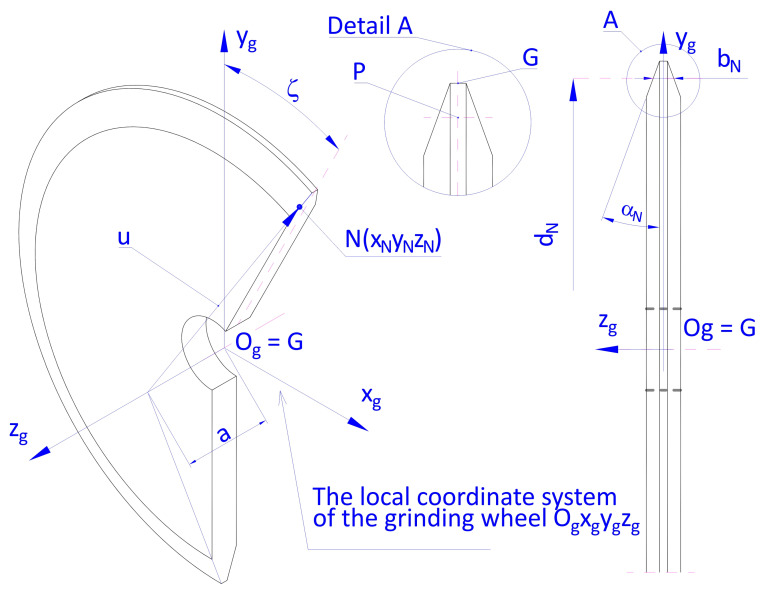
Diagram for the calculation of the side surface of the disk grinding wheel.

The conical-like helical surface is formed as a result of a relative rotational and translational motion of the surface being formed and a rotational motion of the grinding wheel with a trapezoidal axial profile. During machining, the abrasive wheel axis is tilted in relation to the worm axis by the pitch angle of the helical line *γ*_N_ on a cylinder with a specified diameter (usually pitch diameter). By selecting the tilting angle of the abrasive wheel axis *γ*_N_, one may exert influence on the values of deviations from the rectilinearity of the worm axial profile on the apex and on the thread base. The helical surface of the worm is an envelope of the conical surface of the tools as a result of relative helicoidal movement. The parameters that describe the surface of the tool in conical shape, presented in Figure 7, in a local system of coordinates O_g_x_g_y_g_z_g_ will be as follows:

(12) $$N^{\prime }\;=\;\left[\begin{array}{c} x_{N^{\prime }}\\ y_{N^{\prime }}\\ z_{N^{\prime }} \end{array}\right]\;=\;\left[\begin{array}{c} u\cdot cos\;\alpha_{N}\cdot sin\;\zeta \\ u\cdot cos\;\alpha_{N}\cdot cos\;\zeta \\ a-u\cdot sin\;\alpha_{N} \end{array}\right]$$

where u is the distance of point *N′(x_N′_y_N′_z_N′_)* from the cone apex, *α_N_* is the cone profile angle, and *ζ* is the angle of rotation in relation to axis *z_g_*.

The height of the cone a of the side surface of the tool may be calculated from the following formula:

(13) $$a\;=\;\frac{d_{N}}{2}\cdot tan\;\alpha_{N}\;+\;\frac{b_{N}}{2}$$

where *d_N_* is the pitch diameter of the tool, and *b_N_* is the width of the grinding wheel on the diameter corresponding to the worm pitch diameter: $\frac{d_{N}}{2}\;-\;2\cdot m$.

In the absolute system of coordinates Oxyz—in the part of the OUPN system: grinder base *b*, tool headstock sled *c*, tool headstock *d*, abrasive wheel spindle *w* and abrasive wheel *g*—the position of point *N* is described with vector ${\vec{r}}_{N}$, which may be calculated from the following formula:

(14) $$N={\vec{r}}_{N}={\vec{r}}_{b}+{\vec{r}}_{t}+{\vec{r}}_{c}+{\vec{r}}_{d}+{\vec{r}}_{w}+{\vec{r}}_{N^{\prime }}$$

In the calculations above the sum of the vectors there occur rotation matrices that determine the rotation of the local systems of coordinates related to the grinder base *b*, grinder table *t*, tool headstock sled *c*, tool headstock d and the abrasive wheel spindle in relation to their axes and translation matrices, which describe linear shifts in the local systems of coordinates.

The worm helical surface machined with a disk tool, which is an envelope of the tool active surface, which is in helicoidal movement, one that is concentric with the helical surface axis, is not a ruled surface in a general case. In order to explicitly determine such a helical surface, the following is to be provided: the rated profile and dimensions of the abrasive wheel and its situation in relation to the helical surface. In order to generate a general equation of a helical surface machined with a disk tool, the following can be used: the envelope theory, the division method of the disk tool into an infinite number of elementary tools with an infinitely small width or the determination method of the family of the lines of the contact between the helical surface and the tool.

**Figure 8:**
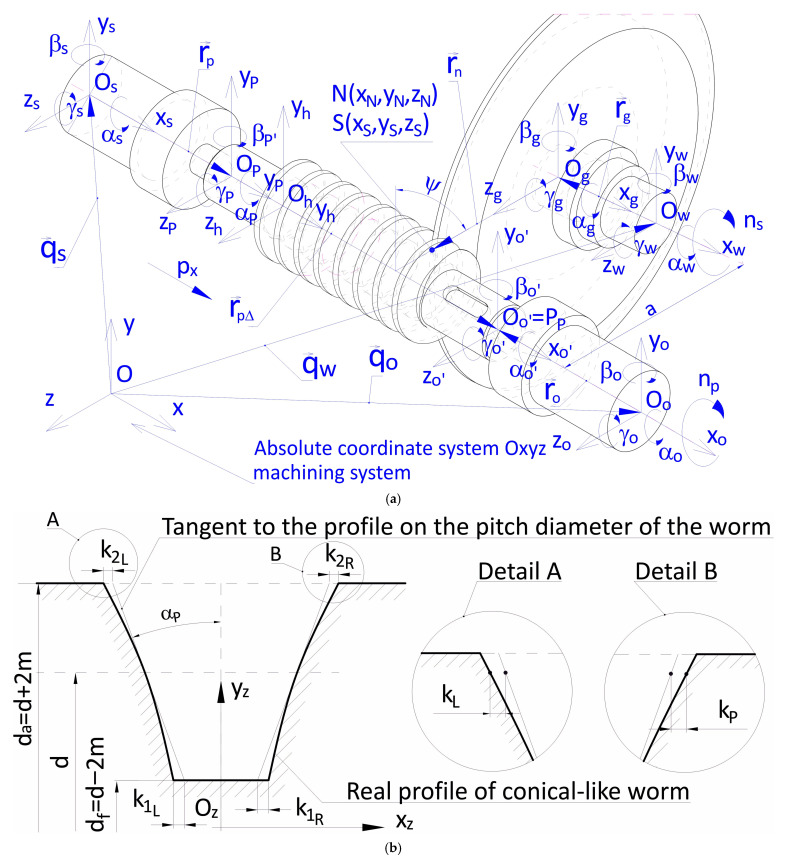
Shaping of the cone-derived helical surface of the worm: (**a**) kinematic system, (**b**) axial profile [18].

What was used for the purpose of further considerations was the relationships in the Oxyz coordinate system accepted and in the local coordinate systems, presented in detail in Figure 8.

In the coordinate system *Ox_s’_y_s’_z_s’_* of the workpiece, the coordinates of the contact line between the tool and the helical surface of the worm are obtained based on the perpendicularity condition of the normal vector ${\vec{n}}_{n}$ to the surfaces in contact and the relative velocity vector ${\vec{\vartheta }}_{n}$ in helicoidal movement.

(15) $${\vec{n}}_{N}\cdot {\vec{\vartheta }}_{N}=0$$

In Dependence (15), the components of the normal vector can be computed based on Equation (12), which describes the tool surface as the adequate determinant in relation to the conical surface parameters u and ζ. The components of the tangent vector are calculated based on the vector of the relative velocity of the tool shift in relation to the worm being formed. By performing the calculations, equations are obtained which, together with Dependence (12), form a system of equations that describe the contact line of the tool surface and the worm in the tool’s coordinate system *Ogxgygzg*. The worm helical surface is formed as a result of a rotation by angle *ψ* and the corresponding shift, where *p* is the pitch of the helical surface.

The final form that describes the conical-like helical surface of the worm tooth in the local coordinates system of the workpiece O_s’_x_s’_y_s’_z_s’_ in a general notation is as follows:

(16) $$S^{\prime }=\left[\begin{array}{c} x_{S^{\prime }}=f_{x}(u,b_{N},d_{N},p,\alpha_{N},\gamma ,{\Delta \gamma }_{N},\zeta ,\psi ,{∆a}_{N})\\ y_{S^{\prime }}=f_{y}(u,b_{N},d_{N},p,\alpha_{N},\gamma ,{\Delta \gamma }_{N},\zeta ,\psi ,{∆a}_{N})\\ z_{S^{\prime }}=f_{z}(u,b_{N},d_{N},p,\alpha_{N},\gamma ,{\Delta \gamma }_{N},\zeta ,\psi ,{∆a}_{N}) \end{array}\right]$$

**Figure 9:**
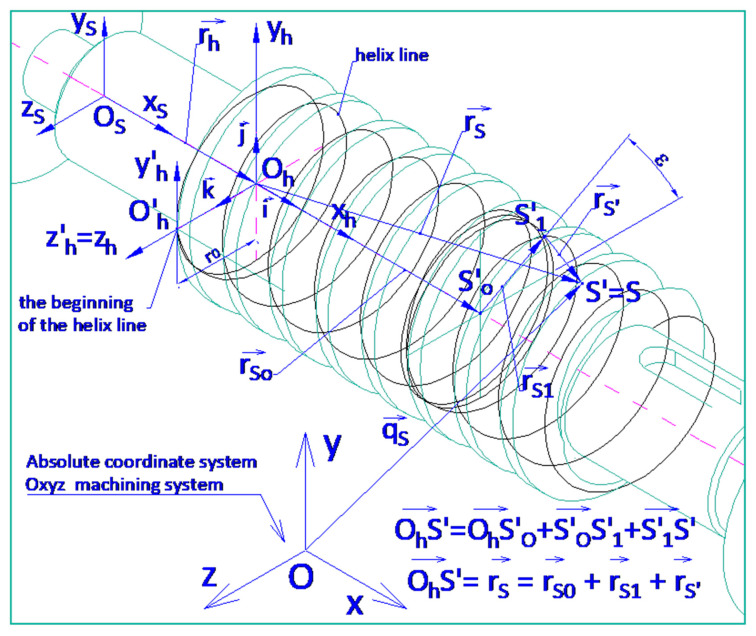
Conical-like helical surface of single-thread worm.

In a general case, the equation was derived of the helical surface machined with a disk tool, where the deviations of the geometric parameters that occur in Equation (17) amount to zero. The vector ${\vec{r}}_{S}$ of any helical surface point (Figure 9) is the sum of vectors ${\vec{r}}_{S_{0}}$, ${\vec{r}}_{S_{1}}$, ${\vec{r}}_{S^{\prime }}$:

(17) $${\vec{r}}_{S}={\vec{r}}_{S_{0}}+{\vec{r}}_{S_{1}}+{\vec{r}}_{S^{\prime }}$$

The vector ${\vec{r}}_{S_{0}}$ is the shift in the movable system O′_h_x′_h_y′_h_z′_h_ in relation to the local system of coordinates O_h_x_h_y_h_z_h_ in parallel to the helicoid axis.

The vector ${\vec{r}}_{S_{1}}$ is the result of the shift in the movable system O′_h_x′_h_y′_h_z′_h_ in relation to the local system of coordinates O_h_x_h_y_h_z_h_ by the quantity of the shift r_0_ and rotation of the system O′_h_x′_h_y′_h_z′_h_ in relation to the system O_h_x_h_y_h_z_h_ by angle ε. The vector ${\vec{r}}_{S^{\prime }}$ is the radius vector of the helical surface profile.

Based on the considerations above, the following equation was derived of the helical surface in the local system of coordinates O_h_x_h_y_h_z_h_:

(18) $$\left.\begin{array}{c} \begin{array}{c} x_{S^{\prime }}=u\cdot \left(\sin\alpha_{N}\cdot \cos\gamma_{N}+\cos\alpha_{N}\cdot \sin\gamma_{N}\cdot \sin\xi \right)-\frac{p_{x}\cdot \psi }{2\cdot \pi }-a\cdot \cos\psi \\ y_{S^{\prime }}=u\cdot \left(-\cos\alpha_{N}\cos\xi \sin\psi +\cos\alpha_{N}\cos\gamma_{N}\sin\xi \cos\psi -\sin\alpha_{N}sin\gamma_{N}cos\psi \right)+\\ +a_{N}\cdot cos\gamma_{N}-a_{N}\cdot \sin\psi \end{array}\\ z_{S^{\prime }}=u\cdot \left({cos\alpha }_{N}\cdot \cos\xi \cdot \cos\psi +\cos\alpha_{N}\cdot cos\gamma_{N}\cdot \sin\xi \cdot \sin\psi \right)+\\ +a_{N}\cdot \sin\gamma_{N}+a_{N}\cdot cos\psi \\ u=a_{N}\cdot \sin\alpha_{N}-\left(a_{N}\cdot \sin\alpha_{N}\cdot \tan\gamma_{N}+\frac{p_{x}}{2\cdot \pi }\cdot \sin\alpha_{N}\right)\cdot \tan\xi +\\ -\frac{\left(a_{N}-\frac{p_{x}}{2\cdot \pi }\cdot \cot\gamma_{N}\right)}{cos\xi }\cdot \cos\alpha_{N} \end{array}\right\}$$

#### 2.3.2. Grinding Accuracy of the Helical Surface of the Worm

For the proper grinding process of helical surfaces, it is necessary to solve not only geometric problems but also numerous technological problems.

The helical surface formed in the grinding process with a disk grinding wheel (Figure 7 and Figure 8) is an envelope of the active surface of the tool that is traveling in relation to it in helicoidal movement.

For the purpose of an explicit determination of the helical surface being machined with a disk tool, the following is to be provided:

- Tool type;
- Tool-rated profile;
- Tool dimensions;
- Position of the tool in relation to the helical surface axis.

A number of factors have an impact on the accuracy of the pitch quality of the thread helical surface; these may be classified as follows:

- Causes that are characteristic of grinders for threads: deviations of the pitch of the lead screw, non-rectilinearity of the table movement, deviations of the shape and position, inaccuracies of the guides, kinematic deviations of the drives;
- Causes that are variable during machining and that are dependent on the machine tool: thermal deformations of the bed and table, thermal deformations of the lead screw, thermal expansion of the grinding wheel spindle, shape-related wear of the grinding wheel, elastic strains of the machine tool;
- Causes that are variable during machining and that are dependent on the workpiece: thermal deformations of the workpiece, elastic strains of the workpiece by machining forces, deviations to the workpiece position.

It was demonstrated that a deviation of the distance of the workpiece axis and the grinding wheel axis Δa_N_ and a deviation of the distance between dressing wheel blades is the cause of the dimensional deviations of the helical surface being machined. A deviation of the grinding wheel tilt angle Δγ_N_, a deviation of the tilt angle of the dressing wheel head Δγ_obc_ = β and a deviation of the grinding wheel profile angle Δα_Nobc_ are the causes of the shape deviations of the helical surface.

Deviations of the shape of the abrasive wheel active surface and deviations of its position in relation to the grinding of the helical surface are of crucial importance to the machining accuracy. These deviations depend to a significant extent on the inaccuracy in the settings of the machine tool.

Based on the considerations described with Equations (17) and (18), a helical surface equation was derived (vector ${\vec{r}}_{h}$ in the local system of coordinates O_h_x_h_y_h_z_h_), machined with a disk tool. Whole deviations from the nominal position are Δα_N_ and Δa_N_.

(19) $$\left.\begin{array}{c} {\vec{r}}_{h}=\left[x_{N}\left(u,\xi \right)\cdot \cos\left(\gamma_{N}+{∆\gamma }_{N}\right)+\gamma_{N}\left(u,\xi \right)\cdot \sin\left(\gamma_{N}+{∆\gamma }_{N}\right)-P\cdot \frac{\psi }{2\cdot \pi }\right]\cdot \vec{i}+\\ +[-x_{N}\left(u,\xi \right)\cdot \cos\psi \cdot \sin\left(\gamma_{N}+{∆\gamma }_{N}\right)+\gamma_{N}\left(u,\xi \right)\cdot \cos\psi \cdot cos\left(\gamma_{N}+{∆\gamma }_{N}\right)+\\ -z_{N}\left(u,\xi \right)\cdot \sin\psi -\left(a_{N}+{∆a}_{N}\right)\cdot \sin\psi ]\cdot \vec{j}+\\ +[-x_{N}\left(u,\xi \right)\cdot \sin\psi \cdot \sin\left(\gamma_{N}+{∆\gamma }_{N}\right)+\gamma_{N}\left(u,\xi \right)\cdot \sin\psi \cdot cos\left(\gamma_{N}+{∆\gamma }_{N}\right)+\\ +z_{N}\left(u,\xi \right)\cdot \cos\psi +\left(a_{N}+{∆a}_{N}\right)\cdot \cos\psi ]\cdot \vec{k}\\ {\vec{n}}_{N}\cdot {\vec{\vartheta }}_{N}=0 \end{array}\right\}$$

whereas

(20) $${\vec{\vartheta }}_{N}={\vec{\omega }}_{N}\cdot {\vec{r}}_{N}+\vec{R}\cdot \vec{\omega }+P\cdot \frac{\vec{\omega }}{2\cdot \pi }$$

(21) $${\vec{n}}_{N}=\left\|\begin{array}{ccc} {\vec{i}}_{N} & {\vec{j}}_{N} & {\vec{k}}_{N}\\ \frac{{\delta x}_{N}}{\delta u} & \frac{{\delta y}_{N}}{\delta u} & \frac{{\delta z}_{N}}{\delta u}\\ \frac{{\delta x}_{N}}{\delta \xi } & \frac{{\delta y}_{N}}{\delta \xi } & \frac{{\delta z}_{N}}{\delta \xi } \end{array}\right\|$$

(22) $$\vec{\omega }=-\omega \cdot \cos\left(\gamma_{N}+{∆\gamma }_{N}\right)\cdot {\vec{i}}_{N}+\omega \cdot \sin\left(\gamma_{N}+{∆\gamma }_{N}\right)\cdot {\vec{j}}_{N}$$

(23) $$\vec{R}=-\left(a_{N}+{∆a}_{N}\right)\cdot {\vec{k}}_{N}$$

In a particular case, a conical-like helical surface, the following set of equations was obtained:

(24) $$\left.\begin{array}{c} \begin{array}{c} x_{S^{\prime }}=u\cdot \left(\sin{\alpha^{\prime }}_{N}\cdot \cos{\gamma^{\prime }}_{N}+\cos{\alpha^{\prime }}_{N}\cdot \sin{\gamma^{\prime }}_{N}\cdot \sin\xi \right)-\frac{p_{x}\cdot \psi }{2\cdot \pi }-a^{\prime }\cdot \cos\psi \\ y_{S^{\prime }}=u\cdot \left(-\cos{\alpha^{\prime }}_{N}\cos\xi \sin\psi +\cos{\alpha^{\prime }}_{N}\cos{\gamma^{\prime }}_{N}\sin\xi \cos\psi -\sin{\alpha^{\prime }}_{N}sin{\gamma^{\prime }}_{N}cos\psi \right)+\\ +a^{\prime }\cdot cos{\gamma^{\prime }}_{N}-{a^{\prime }}_{N}\cdot \sin\psi \end{array}\\ z_{S^{\prime }}=u\cdot \left({cos\alpha^{\prime }}_{N}\cdot \cos\xi \cdot \cos\psi +\cos{\alpha^{\prime }}_{N}\cdot cos{\gamma^{\prime }}_{N}\cdot \sin\xi \cdot \sin\psi \right)+\\ +{a^{\prime }}_{N}\cdot \sin{\gamma^{\prime }}_{N}+{a^{\prime }}_{N}\cdot cos\psi \\ u=a^{\prime }\cdot \sin{\alpha^{\prime }}_{N}-\left({a^{\prime }}_{N}\cdot \sin{\alpha^{\prime }}_{N}\cdot \tan{\gamma^{\prime }}_{N}+\frac{p_{x}}{2\cdot \pi }\cdot \sin{\alpha^{\prime }}_{N}\right)\cdot \tan\xi +\\ -\frac{\left({a^{\prime }}_{N}-\frac{p_{x}}{2\cdot \pi }\cdot \cot{\gamma^{\prime }}_{N}\right)}{cos\xi }\cdot \cos{\alpha^{\prime }}_{N} \end{array}\right\}$$

where

(25) $$a^{\prime }=d_{N}\cdot {tg\alpha^{\prime }}_{N}+\frac{b_{N}+{∆b}_{N}}{2}$$

(26) $${\alpha^{\prime }}_{N}=\alpha_{N}+{∆\alpha }_{N};\;{\gamma^{\prime }}_{N}=\gamma_{N}+{∆\gamma }_{N};\;{a^{\prime }}_{N}=a_{N}+{∆a}_{N}$$

Δα_N_, Δγ_N_, Δa_N_, Δb_N_ are the deviations of the following respectively: abrasive wheel axial profile angle, abrasive wheel axis tilt angle, distance of the abrasive wheel axis from the helical surface axis and the abrasive wheel width on the pitch diameter.

The same surface is described with vector S that determines its location in the absolute system Oxyz of the OUPN process system:

(27) $$S=f\left(R_{S},T_{S},S^{\prime }\right)=\left[\begin{array}{c} x_{S}=f_{x_{S}}\left(R_{S},T_{S},S^{\prime }\right)\\ y_{S}=f_{y_{S}}\left(R_{S},T_{S},S^{\prime }\right)\\ z_{S}=f_{z_{S}}\left(R_{S},T_{S},S^{\prime }\right) \end{array}\right]$$

where R_S_ (R_b_, R_t_, R_c_, R_d_, R_w_, R_h_, R_s_, R_k_, R_o_, R_p_) are the rotation matrices that determine the rotation of the local systems of coordinates related with base b, grinder table t, sled of the tool headstock c, tool headstock d, abrasive wheel spindles w, headstock PO h, spindles PO s, knight k and its axis o.

T_N_ (T_b_, T_t_, T_c_, T_d_, T_w_, R_h_, R_s_, R_k_, R_o_, R_p_) are the translation matrices that describe linear shifts in the local coordinate systems of the process system elements.

An analytical description of the configuration of the OUPN kinematic system comprises the transformations of Equations (8)–(27) until those dependences have been obtained that determine the angular and linear parameters. However, it needs to be emphasized that for the present OUPN process system, entangled dependences are obtained based on which the coordinates of the surface or only the profile (e.g., axial profile) can be determined using numerical methods. The coordinates of the helical surface can also be determined through logical operations on a set of solids in the CAD environment.

**Figure 10:**
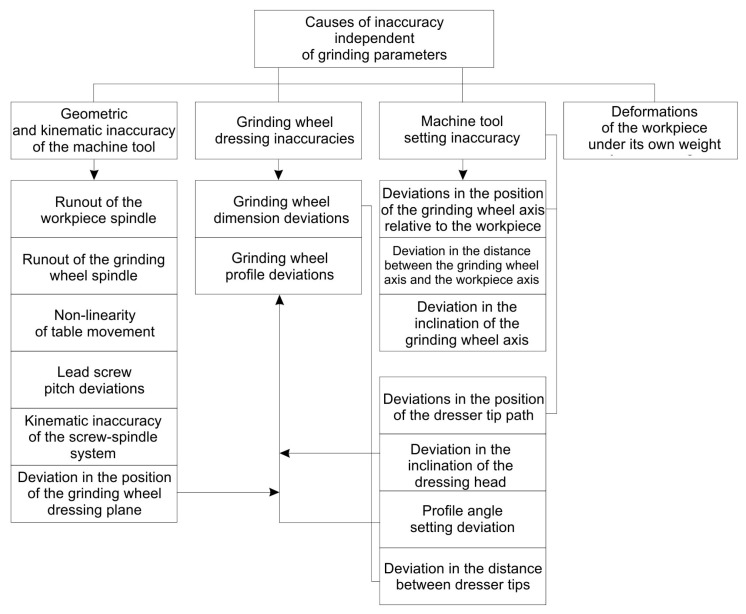
Causes of inaccuracies in the helical surface grinding process.

Analysis of machining accuracy in the technological system (Figure 10) indicates that the main causes of profile deviations can be attributed to three direct causes:

Deviations in the position of the grinding wheel relative to the axis of the workpiece [28];

Deviations in the inclination angle of the grinding wheel axis relative to the axis of the machined helical surface;

Deviations in the grinding wheel profile.

The profile deviation of the grinding wheel can be caused by inaccuracies in the profiling device and deviations in its position, but the main reason is shape wear during machining. Geometric accuracy deviations and deformations of machine tools cause profile deviations of less than 0.001 mm. The inclination angle deviation of the grinding wheel axis significantly affects the profile deviation value. The mechanisms for setting the inclination angle of the grinding wheel axis require improvements or the use of additional measuring instruments.

### 2.4. Design Methods for Enhancing Gear Accuracy

**Figure 11:**
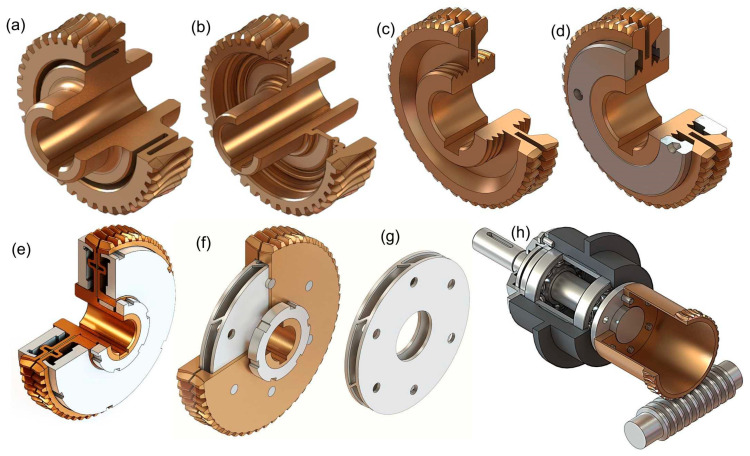
Design variants of worm wheels for periodic backlash adjustment in worm gear transmissions (**a**–**h**).

Gears capable of ensuring backlash-free operation of a technical system are considered special gears [12,29]. They can differ in the type and shape of the interacting surface elements [13]. They also vary in methods for reducing the groove width in worm wheels and solutions that allow for reducing the axial pitch of the worm without disassembling the gear [18]. A mechanism that allows changing the distance between the axes of the worm and the worm wheel can be used to minimize side backlash. Another solution is a gear in which the worm wheel has a rim that can move elastically relative to the hub (Figure 11) or has a peripheral slot, the width of which can be reduced by deforming the separated sides of the rim, or the worm wheel is divided into two parts [18], with the division surface defined by a plane passing through the center of the worm wheel and perpendicular to its axis of rotation. Both parts are mounted on a common hub. Angular displacement of one half relative to the other allows increasing the thickness of the worm wheel teeth, for example, through axial compression of a disk with oblique ribs. Examples of such design solutions are presented in Figure 11, which illustrates several variants of worm wheels intended for periodic backlash adjustment. Worm wheel with an axially flexible diaphragm-type connection between the hub and the rim (Figure 11a); worm wheel with a radially corrugated wall connecting the rim with the hub (Figure 11b); worm wheel with a circumferential deep groove providing increased axial compliance of both sides of the rim (Figure 11c); worm wheel with conical clamping of both sides of the rim, ensuring reduction in the groove width and bringing the outer parts of the teeth closer together (Figure 11d); worm wheel with tensioning disks providing elastic support of the rim (Figure 11e); worm wheel composed of two disk-shaped parts connected by a plate in the form of two circular segments joined by inclined ribs, which causes a slight relative rotation of the worm wheel halves as a result of their axial compression, leading to an increase in tooth thickness and thus reduction in backlash (Figure 11f); view of the plate for the gear shown in Figure 11f (Figure 11g); worm wheel with a toothed rim mounted on a thin compliant sleeve.

When evaluating different design solutions, the following criteria can be used, representing the following requirements:

- A wide range of side backlash adjustment;
- The ability to adjust backlash without the need to disassemble gear components;
- Self-minimization of backlash within a certain range of regulatory settings;
- A positive impact on gear durability;
- Low production costs;
- Gear sizes not exceeding those of conventional designs;
- Reduction in dynamic reactions to load changes;
- Absence of local increases in pressure on interacting surfaces.

**Figure 12:**
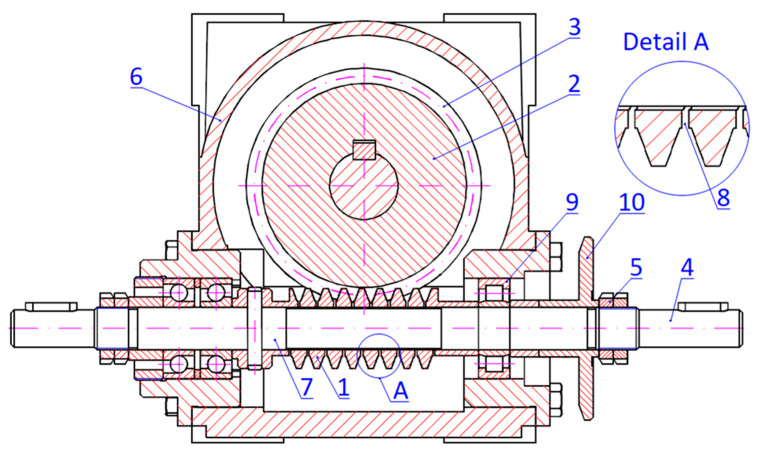
Gear with a worm locally compliant in the axial direction. Construction worm gear drive with a locally axially adaptive worm: 1—worm, 2—worm wheel’s rim, 3—worm wheel 4—spindle, 5—pressure nuts, 6—housing, 7—fitted worm situation surface, 8—helical cuts, 9—roller bearing, 10—displacement measurement plate [18].

The conclusion from the analysis of the features of various designs is as follows [25]—the solution meeting most of the requirements is the gear with an axially compliant worm (Figure 2 and Figure 12). Figure 12 shows the design idea of the worm gear with an axially flexible worm. This flexibility is achieved by making a careful change in the design of the thread roots, thus enabling backlash control by axial movement. Flexibility in this case is achieved through a local change in the structure, namely, by cutting a slot at the bottom of the threads to form a region of elasticity.

The rolling bearing (9) provides adequate support for the moving part, allowing for the necessary movement along the axis of the system. Thus, flexibility is achieved locally and does not significantly reduce the rigidity of the entire system.

**Figure 13:**
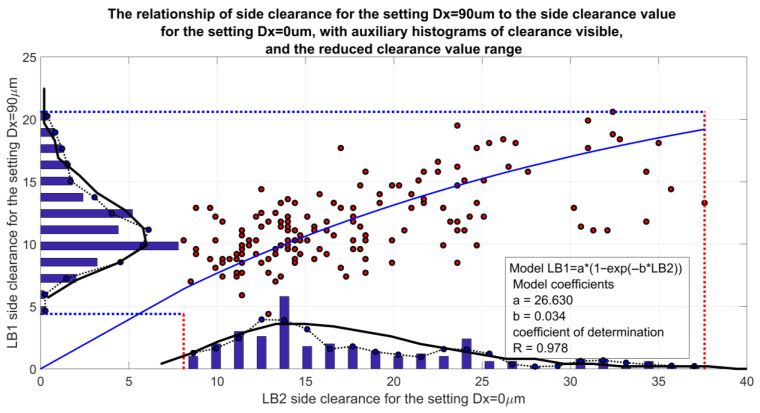
The relationship between backlash after introducing the adjustment setting and the backlash value before adjustment [31].

The range of side backlash adjustment in this gear is considerable, and the adjustment itself is easy because the adjustment mechanism is located outside the housing. The gear features good lubrication of the interacting elements and allows for multiple backlash adjustments in response to the progressive wear of the worm and worm wheel. Introducing an adjustment setting of Dx > 0 causes a reduction in the axial pitch of the worm thread and a decrease in backlash value (Figure 13). Gradually, a state is reached in which, in one of the relative positions of the worm and worm wheel, a determined minimum backlash value is achieved (in Figure 13, this is 5 micrometers). It is also possible to set a zero side backlash value for one of the angular positions. In this state, the force causing axial deformation of the worm does not affect the gear’s motion resistance, which depends solely on the gear load. Beyond this state, a slight increase in the setting (axial compression of the worm) is permissible, but it is limited to a few micrometers to avoid increasing the gear’s motion resistance. Reducing backlash to negative values in a specific position increases motion resistance due to local deformation of the worm wheel rim.

**Figure 14:**
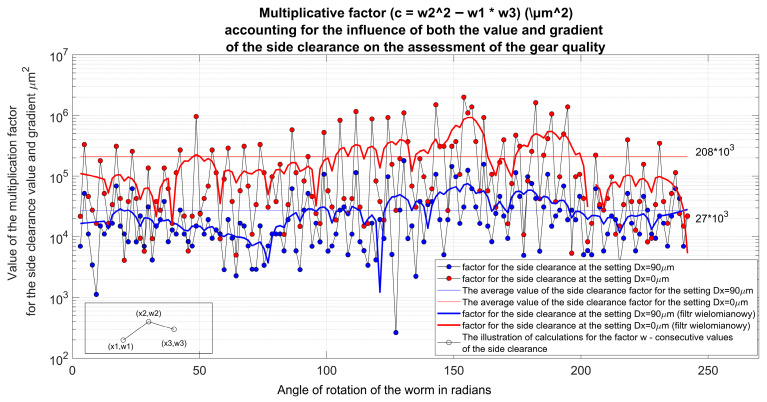
Multiplicative quality indicator for worm gears [31].

The quality assessment of the gear can be conducted based on an indicator that incorporates the product of the local backlash value and the absolute value of the backlash gradient (Figure 14). The variables w_1_, w_2_, and w_3_ refer to the clearance value taken at three consecutive angles of rotation of the worm and are used to calculate the variance in clearance by means of the coefficient c. The usefulness of this indicator arises from the fact that large backlash values and significant gradient values are unfavorable in a given position of the interacting elements. From the presented results, it can be concluded that the studied design solution with the described backlash adjustment significantly enhances the quality of the gear. Further beneficial effects can be achieved by optimizing the assembly of components: the worm, worm wheel, and gear housing. Additionally, this described design allows for the introduction of additional material into the gap formed after cutting the root of the worm thread, to beneficially impact vibration damping or to increase the load capacity of the gear by stiffening the worm.

### 2.5. Procedures for Optimal Assembly of Precision Worm Gears in Small Batch Production for Various Accuracy Evaluation Criteria of the Entire Series

The procedures discussed in this section are based on the algorithmic structure and optimization approach developed in the current research. Therefore, the component assembly approaches discussed here must be considered a direct result of the chosen mathematical model for backlash formation and variance. These procedures provide a structured approach to choosing and matching gears to minimize backlash and variance in small batches. The backlash in the meshing is caused by deviations in the gear elements such as worm thread lead, worm thread pitch, worm thread thickness, worm thread runout, worm thread profile, worm wheel teeth circumferential pitch, worm wheel teeth runout, worm wheel teeth thickness and distance between worm and worm wheel axes. The influence of these deviations is dependent on their components in the circumferential direction of the worm wheel and the axial direction of the worm. In most cases where more than one worm wheel tooth is in mesh, the assessment of the influence of each characteristic must take into account the amplitude of the envelope of their instantaneous values, which are locally variable. For the analyses related to the selection of the method to assemble the gear elements the following criteria to optimize the entire production series of gears were used:

- Minimum of the maximum backlash value in the set of maximum values for each gear;
- Minimum range of backlash values in the set of maximum values for each gear;
- Minimum standard deviation of backlash values in the set of maximum values for each gear;
- Minimum of the product of the maximum value and the standard deviation of backlash for all gears;
- Minimum of the maximum product of the backlash gradient and backlash value in the set of maximum values for each gear in the entire production series;
- Maximum arithmetic mean of backlash values in the set of maximum values for each gear in the entire production series (maximizing the arithmetic mean means minimizing the variation in backlash values among individual gears).

Assembling gears from elements with different accuracy without optimization results in gears where kinematic deviation (backlash) can be very variable, which means big quality differences. When the quality of the entire series is determined by the worst result it lowers the production quality rating. Here are the optimization results for assembling precision gears with an axially compliant worm to find a set of assemblies where the level of differentiation of the maximum backlash value for each gear is minimal. In the further analysis, each gear will be characterized by the maximum backlash value in the meshing, which is usually at one angular position of the worm and worm wheel. Therefore the set of backlash values for all gears will contain the maximum value for each of them. For simplicity these values will be referred to as gear backlash. The influence of the worm, worm wheel, and gear housing on the backlash was determined using the calculation procedure based on the adopted geometrical and technological model. The effects of deviations such as worm thread lead deviation, worm thread pitch deviation, worm thread thickness deviation, worm thread runout, worm thread profile deviation, worm wheel teeth circumferential pitch, worm wheel teeth runout, worm wheel teeth thickness deviation and deviation of the distance between worm and worm wheel axes a_w_ were calculated on the basis of the mathematical relationships given in Equations (19)–(27). These deviations affect the creation of backlash to different extents depending on their components in the circumferential direction of the worm wheel, i.e., in the axial direction of the worm. It was considered that when more than one worm wheel tooth is in mesh at a given angular position, the backlash in the mesh is the value of the envelope of locally variable instantaneous values. The individual deviations of the gear element characteristics under control such as worm thread lead deviation, worm thread pitch deviation, worm thread thickness deviation, worm thread runout, worm thread profile deviation, worm wheel teeth circumferential pitch, worm wheel teeth runout, worm wheel teeth thickness deviation and deviation of the distance between worm and worm wheel axes affect the creation of backlash to different extents depending on their components in the circumferential direction of the worm wheel and the axial direction of the worm. Moreover when more than one worm wheel tooth is in mesh at a given position the backlash in the mesh is the envelope of locally variable instantaneous values. The accuracy of the worm is mainly determined by the last operation, which is grinding the thread surface. The factors that most affect the backlash value are lead, profile, thread thickness deviations and radial runout of the thread. Usually the accuracy of worm wheels is lower than the accuracy of worms. The teeth of worm wheels are usually shaped during hobbing. The factors that most affect the deviations of the tooth surfaces are then the kinematic accuracy deviations of the machine tool, the positioning deviations of the worm wheel and tool and the geometric deviations of the hobs caused by uneven wear of the cutting edges or their sharpening. Therefore, the factors that most affect the accuracy of the worm wheel are tooth thickness deviations, circumferential pitch deviations, tooth profile deviations and tooth runout. A common deviation is the runout of the worm wheel teeth caused by the deviation of its mounting on the locating pin.

## 3. Results

**Figure 15:**
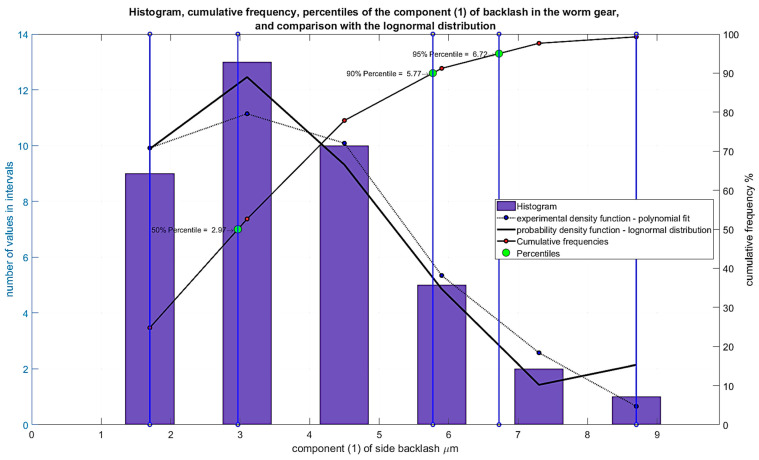
Histogram of Component (1) of backlash resulting from dimensional and shape deviations of the worm helical surface.

**Figure 16:**
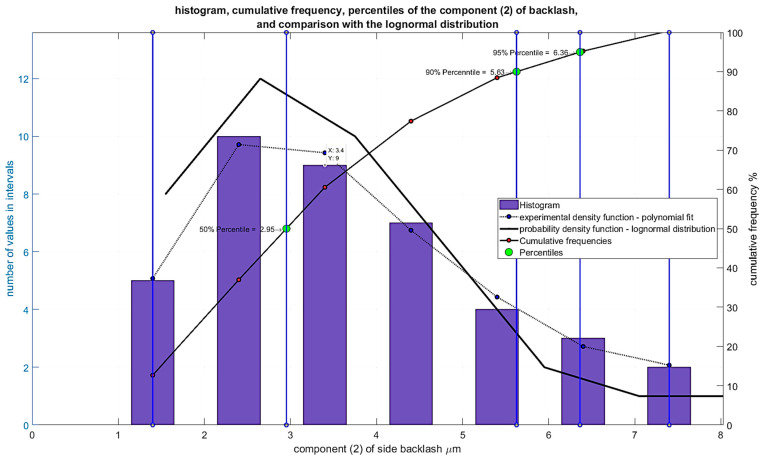
Histogram of Component (2) of backlash resulting from dimensional and shape deviations of the worm wheel teeth surface.

**Figure 17:**
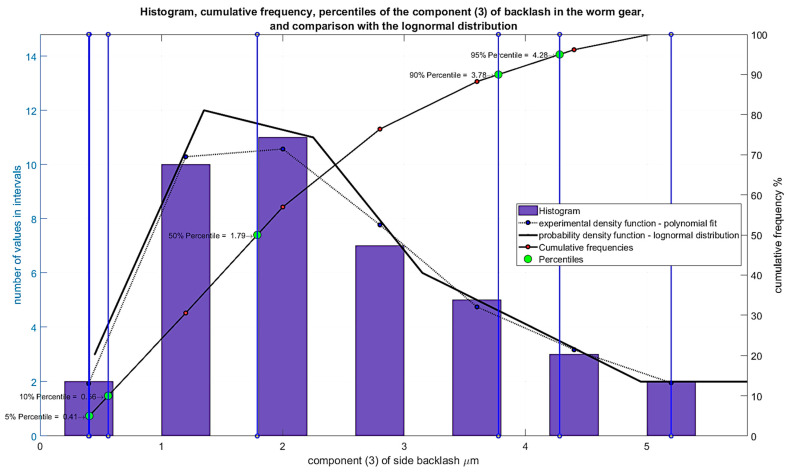
Histogram of Component (3) of backlash resulting from deviations in the distance between the axes of the worm and worm wheel, due to housing dimensional deviations.

The results of the analyses are presented for the features of the component elements outlined below. It was assumed that the cooperating elements of the gear unit have varied accuracy deviations, which, after calculating their impact on backlash, had values presented in the form of histograms in Figure 15, Figure 16 and Figure 17.

Dimensional and shape deviations of the worm helical surface, recalculated to their impact on backlash maximum values for each worm in the series (40), are expressed in micrometers (Figure 15)—Component (1).

Dimensional and shape deviations of the worm wheel teeth surface, recalculated to their impact on backlash maximum values for each worm wheel in the series (40), are expressed in micrometers (Figure 16)—Component (2).

Deviations in the distance between the axes of the worm and worm wheel, resulting from deviations in the dimensions of the housing, recalculated to their impact on backlash—maximum values for each housing in the series (40)—are expressed in micrometers (Figure 17)—Component (3).

The following criteria were highlighted for evaluating gear accuracy in small-batch production, for which optimization calculations were performed:

- Minimum of the maximum (M) backlash value in the set of maximum values for each gear unit.
- Minimum range (R) of backlash values in the set of maximum values for each gear unit.
- Minimum standard deviation (S) of backlash values in the set of maximum values for each gear unit.
- Minimum product of range (R) and standard deviation (S) of backlash (R × S) in the set of all gear units.

**Figure 18:**
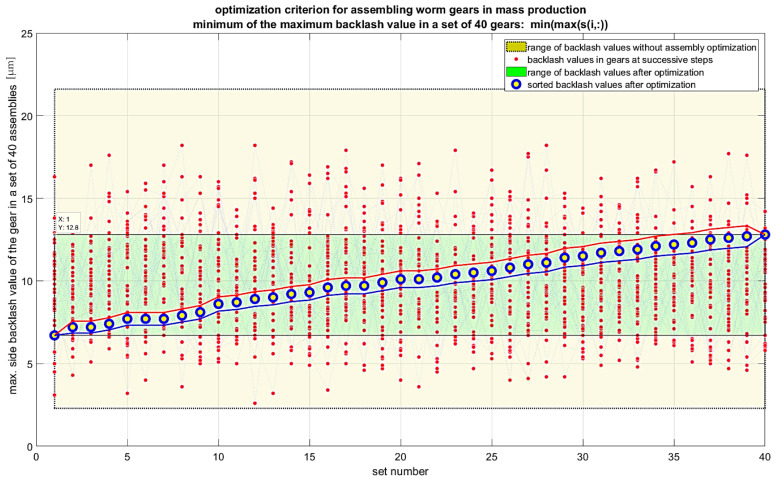
Optimization results for gear assembly according to the criterion of minimizing the maximum backlash value of individual gears.

**Figure 19:**
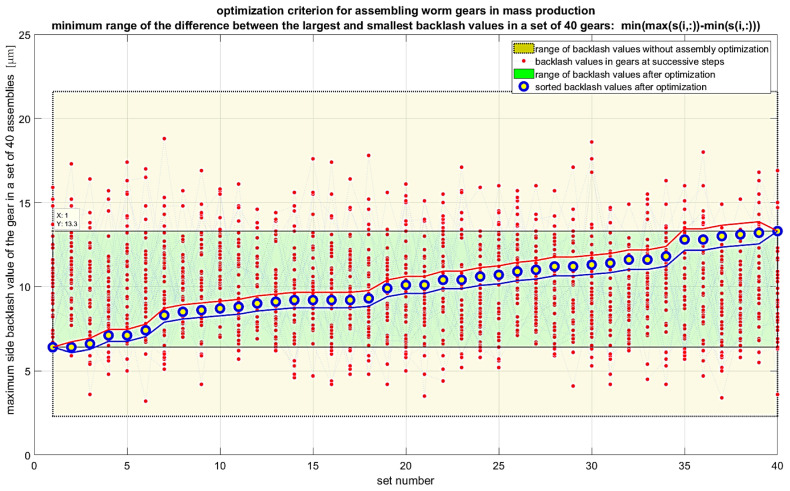
Results of gear assembly optimization according to the criterion of minimizing the range of backlash values of individual gears.

**Figure 20:**
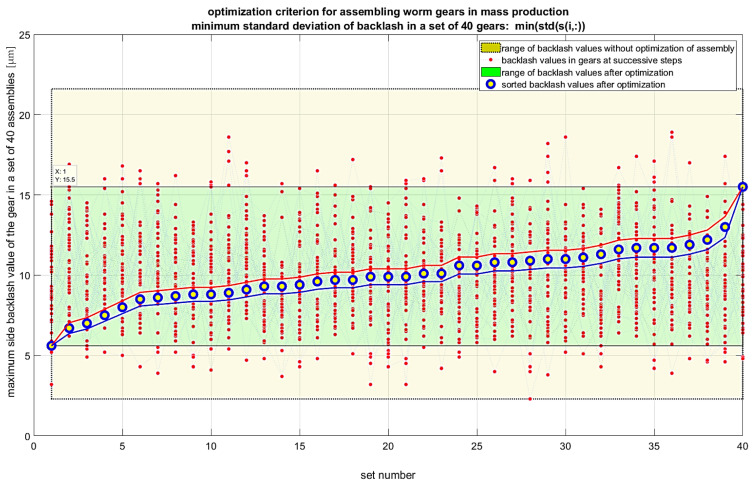
Results of optimization of gear assembly selection for the criterion of minimizing the standard deviation of side backlash values in individual gear units.

**Figure 21:**
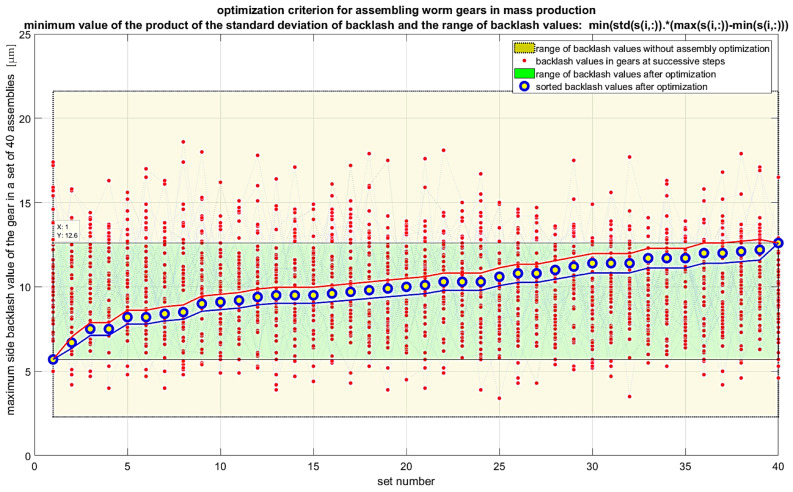
Results of gear assembly optimization according to the criterion of minimizing the product of the standard deviation and the range of backlash values of individual gears.

It is also possible to extend the analyses to include criteria that consider the gradient of backlash values. In this case, the first derivative of backlash for a given set of angular positions of the worm and worm wheel should be considered. These positions are determined by the chosen step for measuring backlash values, for example, for *k* points per revolution of the worm, which gives *k × z*_2_ points for a full revolution of the worm wheel with z_2_ teeth. In such a case, the recommended optimization criterion would be the minimum of the maximum value of the product of the backlash gradient and the backlash value in the set of maximum values for each gear unit in the entire production series. Another optimization criterion can be the maximum of the geometric mean of the backlash values in the set of maximum values for each gear unit in the entire production series. For this criterion, the maximum value should be sought, as maximizing the geometric mean reduces the variability of backlash values across different gear units. Various algorithms can be used to solve the optimization problem. Random sampling algorithms without replacement are inefficient as they generate many repetitive results. Genetic algorithms are recommended for large datasets. Permutation algorithms for small datasets allow calculating and visualizing all cases within a few seconds, which makes their use preferable. The results of the calculations for individual criteria are presented in Figure 18, Figure 19, Figure 20 and Figure 21. Blue markers with yellow filling represent the best-found solution for the given criterion. Markers with red indicators show subsequent better solutions leading to improved results in subsequent steps. Lines connecting the data values are included only to better indicate the subsequent solutions in each step. The conducted optimization of gear component selection indicates that without optimization, the backlash values in the entire series can range from 2 to 22 micrometers, meaning a range of 20 micrometers. For the optimization criterion, the minimum of the maximum backlash value in the set of maximum values for each gear unit, the backlash range obtained was 6 to 13 micrometers. The range was 7 micrometers, which is 35% of the value without optimization. The standard deviation of backlash values after optimization was 1.78 micrometers. For the optimization criterion, the minimum range of backlash values in the set of maximum values for each gear unit, the backlash range obtained was 1 micrometer larger. The range was about 8 micrometers, which is 40% of the value without optimization. The standard deviation of backlash values after optimization was 1.78 micrometers. For the optimization criterion, the minimum standard deviation of backlash values in the set of maximum values for each gear unit, the backlash range obtained was 6 to 16 micrometers. The range was 10 micrometers, which is 50% of the value without optimization. The standard deviation of backlash values after optimization was 1.81 micrometers. For the optimization criterion—the minimum product of the maximum value and the standard deviation of backlash values in the set of maximum values for each gear unit—the backlash range obtained was 6 to 13 micrometers. The range was 7 micrometers, which is 35% of the value without optimization. The standard deviation of backlash values after optimization was 1.81 micrometers. The conducted optimizations indicate that it is possible to assemble worm gears in a way that achieves similar backlash values for the entire production series. For the most favorable result optimization, for the criterion of minimum standard deviation of backlash values in the set of maximum values for each gear unit, the backlash range obtained was 7 micrometers, and the standard deviation was less than 1.8 micrometers.

The methodology for assembling worm gears can be easily utilized in production to align the range of backlash guaranteed by the manufacturer, resulting in a significant improvement in production quality. The simple permutation algorithm applied can be useful for small datasets. It is recommended to start the calculations from different starting points. The optimization has been illustrated for small-batch production since precision gears are not produced in mass quantities. However, this algorithm, or when supplemented with evolutionary algorithms, can successfully be applied in large-scale production.

## 4. Summary and Conclusions

This publication presents the results of various methods for improving the accuracy of worm gears. Diverse techniques for enhancing the machining processes of worms and worm wheels significantly improve the accuracy of the machined helical surfaces. The grinding processes of worm helical surfaces can be optimized to reduce pitch deviations and profile deviations of the worm thread. The main causes of tooth deviations in worm wheels are also highlighted.

The findings that have been achieved prove the correctness of the assumptions initially made. The developed structural scheme demonstrates better properties of meshing, in particular concerning its transmission errors, proving the validity of the used approach. There are no significant differences from the expected properties within the studied range of parameters. The features of different solutions ensuring favorable design changes are described, leading to reduced backlash and improved kinematic accuracy of the gears. It has been demonstrated that the most advantageous solution for backlash adjustment is a gearbox with an axially flexible worm in the central thread zone. For this solution, the backlash adjustment range is significant, and the adjustment does not require disassembly of the gearbox, as the adjustment mechanism is located outside the housing. Moreover, the zonal flexibility of the worm contributes to a certain degree of self-adaptation in the meshing zone.

The developed algorithm for optimizing gear assembly in small-batch production ensures the selection of gear components based on specified criteria, resulting in a significant improvement in production quality. For the presented data, without optimization of component selection, the backlash values in the entire series of produced gear units can range from 2 to 22 micrometers, giving a range of 20 micrometers. For the presented gear assembly optimization criteria, the ranges obtained were much smaller, between 7 and 10 micrometers. The standard deviation of backlash values after optimization was approximately 1.8 micrometers. An almost threefold reduction in the range of backlash values signifies a substantial improvement in the quality of the produced gear units.

Of all the criteria examined in the analysis, the criterion based on minimizing the standard deviation of backlash values results in the best performance because it provides the highest level of consistency in the accuracy of gear units produced. All criteria mentioned above prove to be equally efficient in minimizing maximum backlash values, but they vary in terms of their ability to restrict the variation in backlash between gear units.

As far as the practical implementation of the suggested criterion is concerned, its application requires measuring and sorting out parts, which can increase assembly time. Still, since such measurements are typical of high precision industries, no additional costs will be incurred to perform the task.

The future direction of research is to generalize the present study to include the dynamic and vibro-acoustic analysis with special emphasis on the correlation between the transmission error and the noise response in the frequency domain. For such an approach, a detailed dynamic analysis of the gearbox or a specialized experimental validation becomes essential.

## Data Availability

The original contributions presented in this study are included in the article. Further inquiries can be directed to the corresponding author.

## Nomenclature

Oxyz	absolute coordinate system of the technological setup
OUPN	machine, object, tool (absolute coordinate system of the technological system)
Ox_s_y_s_z_s_	local coordinate system of the workpiece spindle
Ox_p_y_p_z_p_	local coordinate system of the workpiece—worm
Ox_o_y_o_z_o_	local coordinate system of the grinder tailstock
Ox_o’_y_o’_z_o’_	local coordinate system of the grinder tailstock’s axis
Ox_w_y_w_z_w_	local coordinate system of the grinding wheel spindle
Ox_g_y_g_z_g_	local coordinate system of the grinding wheel
P_x_	longitudinal feed of the workpiece—worm
n_p_	rotational speed of the workpiece—worm
n_s_	rotational speed of the grinding wheel
a	distance between the screw surface axis and the grinding wheel axis
N(x_N_y_N_z_N_)	coordinates describing the surface shape of a conical disk grinding wheel
${\vec{q}}_{s}$	vector defining the position of the local coordinate system Ox_s_y_s_z_s_ relative to the absolute coordinate system Oxyz
${\vec{r}}_{p}$	vector defining the position of the local coordinate system Ox_p_y_p_z_p_ relative to the local coordinate system Ox_s_y_s_z_s_
${\vec{q}}_{o}$	vector defining the position of the local coordinate system Ox_o_y_o_z_o_ relative to the absolute coordinate system Oxyz
${\vec{r}}_{o^{\prime }}$	vector defining the position of the local coordinate system Ox_o’_y_o’_z_o’_ relative to the local coordinate system Ox_o_y_o_z_o_
${\vec{r}}_{p\Delta }$	closing link vector determining the position accuracy of the worm
${\vec{q}}_{w}$	vector defining the position of the local coordinate system Ox_w_y_w_z_w_ relative to the absolute coordinate system Oxyz
${\vec{r}}_{g}$	vector specifying the position of the local coordinate system Ox_g_y_g_z_g_ relative to the local coordinate system Ox_w_y_w_z_w_
${\vec{r}}_{n}$	vector specifying the position of the point N(x_N_y_N_z_N_) relative to the local coordinate system Ox_g_y_g_z_g_
α_s_, β_s_, γ_s_	angular deviation of the axis x_s_, y_s_, z_s_ relative to the local coordinate system Ox_s_y_s_z_s_
α_p_, β_p_, γ_p_	angular deviation of the axis x_p_, y_p_, z_p_ relative to the local coordinate system Ox_p_y_p_z_p_
α_o_, β_o_, γ_o_	angular deviation of the axis x_o_, y_o_, z_o_ relative to the local coordinate system Ox_o_y_o_z_o_
α_o’_, β_o’_, γ_o’_	angular deviation of the axis x_o’_, y_o’_, z_o’_ relative to the local coordinate system Ox_o’_y_o’_z_o’_
α_w_, β_w_, γ_w_	angular deviation of the axis x_w_, y_w_, z_w_ relative to the local coordinate system Ox_w_y_w_z_w_
α_g_, β_g_, γ_g_	angular deviation of the axis x_g_, y_g_, z_g_ relative to the local coordinate system Ox_g_y_g_z_g_
${{\vec{q}}_{P}}_{L}$ $i-{{\vec{q}}_{P}}_{p}$	vectors that determine the position of points P_L_ and P_P_, which determine the position of the workpiece p fastened in w lathe centers, in relation to the global system of coordinates Oxyz
$\delta_{P_{∆}}$	deviation of the angular position of the axis of the workpiece
*γ*_N_	abrasive wheel axis
u	distance of point N′(x_N′_y_N′_z_N′_) from the cone apex
α_N_	cone profile angle
ζ	angle of rotation in relation to axis z_g_
d_N_	pitch diameter of the tool
*b_N_*	width of the grinding wheel on the diameter corresponding to the worm pitch diameter: $\frac{d_{N}}{2}-2\cdot m$
${\vec{n}}_{n}$	normal vector to the surfaces in contact
${\vec{\vartheta }}_{n}$	relative velocity vector in helicoidal movement
ψ	angle of rotation
${\vec{r}}_{S_{0}}$	vector is the shift in the movable system O′_h_x′_h_y′_h_z′_h_ in relation to the local system of coordinates O_h_x_h_y_h_z_h_ in parallel to the helicoid axis
${\vec{r}}_{S_{1}}$	vector is the result of the shift in the movable system O′_h_x′_h_y′_h_z′_h_ in relation to the local system of coordinates O_h_x_h_y_h_z_h_ by the quantity of the shift r_0_ and rotation of the system O′_h_x′_h_y′_h_z′_h_ in relation to the system O_h_x_h_y_h_z_h_ by angle ε
${\vec{r}}_{S}$	vector is the radius vector of the helical surface profile
Δα_N,_ Δγ_N,_ Δa_N,_ Δb_N_	deviations of the following respectively: abrasive wheel axial profile angle, abrasive wheel axis tilt angle, distance of the abrasive wheel axis from the helical surface axis and the abrasive wheel width on the pitch diameter
R_S_ (R_b_, R_t_, R_c_, R_d_, R_w_, R_h_, R_s_, R_k_, R_o_, R_p_)	rotation matrices that determine the rotation of the local systems of coordinates related with the base b, grinder table t, sled of the tool headstock c, tool headstock d, abrasive wheel spindles w, headstock PO h, spindles PO s, knight k and its axis o
T_N_ (T_b_, T_t_, T_c_, T_d_, T_w_, R_h_, R_s_, R_k_, R_o_, R_p_)	translation matrices that describe linear shifts in the local coordinate systems of the process system elements
